# Supplementary material for: Fecal Microbiome Differences in Angus Steers with Differing Feed Efficiencies during the Feedlot-Finishing Phase
Source: Microorganisms. 2022 May 31;10(6):1128. doi: 10.3390/microorganisms10061128 (PMC9227454; doi:10.3390/microorganisms10061128)
Supplement: Supplementary file 1 [file microorganisms-10-01128-s001.zip › microorganisms-1685845-supplementary.pdf]

***Supplemental Table S1.*** *Performance of each animal used in the study during the feedlot-finishing phase. RFI = Residual Feed Intake; DMI = Dry Matter Intake; ADG = Average Daily Gain; FG = Feed to Gain ratio; HCW = Hot Carcass Weight; RIBEYE = Ribeye Area; MARB = Carcass Marbling Score.*

| SampleID | AnimalID | SampleType | RFIStatus | DMI | ADG | FG | RFI | HCW | RIBEYE | MARB |
|---|---|---|---|---|---|---|---|---|---|---|
| 69 | 7024 | OFFTEST | High | 12.10 | 1.02 | 11.92 | 0.30 | 346.73 | 70.79 | 480 |
| 90 | 7024 | ONTEST | High | 12.10 | 1.02 | 11.92 | 0.30 | 346.73 | 70.79 | 480 |
| 70 | 7043 | OFFTEST | High | 13.15 | 0.95 | 13.78 | 0.45 | 391.09 | 88.47 | 570 |
| 91 | 7043 | ONTEST | High | 13.15 | 0.95 | 13.78 | 0.45 | 391.09 | 88.47 | 570 |
| 73 | 7059 | OFFTEST | High | 12.04 | 1.06 | 11.40 | 0.32 | 344.23 | 75.19 | 620 |
| 87 | 7059 | ONTEST | High | 12.04 | 1.06 | 11.40 | 0.32 | 344.23 | 75.19 | 620 |
| 76 | 7135 | OFFTEST | High | 15.30 | 1.13 | 13.56 | 2.44 | 416.62 | 88.11 | 650 |
| 92 | 7135 | ONTEST | High | 15.30 | 1.13 | 13.56 | 2.44 | 416.62 | 88.11 | 650 |
| 77 | 7141 | OFFTEST | High | 12.19 | 0.97 | 12.56 | 0.46 | 332.44 | 70.60 | 730 |
| 88 | 7141 | ONTEST | High | 12.19 | 0.97 | 12.56 | 0.46 | 332.44 | 70.60 | 730 |
| 79 | 7011 | OFFTEST | Low | 10.42 | 1.00 | 10.37 | -0.69 | 345.18 | 74.84 | 580 |
| 89 | 7011 | ONTEST | Low | 10.42 | 1.00 | 10.37 | -0.69 | 345.18 | 74.84 | 580 |
| 72 | 7050 | OFFTEST | Low | 13.07 | 1.48 | 8.81 | -0.78 | 368.09 | 91.24 | 710 |
| 85 | 7050 | ONTEST | Low | 13.07 | 1.48 | 8.81 | -0.78 | 368.09 | 91.24 | 710 |
| 74 | 7112 | OFFTEST | Low | 10.52 | 1.12 | 9.42 | -1.55 | 360.61 | 73.61 | 510 |
| 82 | 7112 | ONTEST | Low | 10.52 | 1.12 | 9.42 | -1.55 | 360.61 | 73.61 | 510 |
| 75 | 7124 | OFFTEST | Low | 10.16 | 0.70 | 14.52 | -1.22 | 421.84 | 76.24 | 670 |
| 83 | 7124 | ONTEST | Low | 10.16 | 0.70 | 14.52 | -1.22 | 421.84 | 76.24 | 670 |
| 78 | 7164 | OFFTEST | Low | 10.31 | 0.78 | 13.22 | -1.19 | 397.07 | 96.36 | 780 |
| 80 | 7164 | ONTEST | Low | 10.31 | 0.78 | 13.22 | -1.19 | 397.07 | 96.36 | 780 |
